# A Linkage-specific Sialic Acid Labeling Strategy Reveals Different Site-specific Glycosylation Patterns in SARS-CoV-2 Spike Protein Produced in CHO and HEK Cell Substrates

**DOI:** 10.3389/fchem.2021.735558

**Published:** 2021-09-24

**Authors:** Qiong Wang, Yan Wang, Shuang Yang, Changyi Lin, Lateef Aliyu, Yiqun Chen, Lisa Parsons, Yuan Tian, Hongpeng Jia, Andrew Pekosz, Michael J. Betenbaugh, John F. Cipollo

**Affiliations:** ^1^ Laboratory of Bacterial Polysaccharides, Division of Bacterial, Parasitic and Allergenic Products, Center for Biologics Evaluation and Research, Food and Drug Administration, Baltimore, MD, United States; ^2^ Mass Spectrometry Facility, National Institute of Dental and Craniofacial Research, National Institutes of Health, Bethesda, MD, United States; ^3^ Center for Clinical Mass Spectrometry, School of Pharmaceutical Sciences, Soochow University, Jiangsu, China; ^4^ Facility for Biotechnology Resources, Center for Biologics Evaluation and Research, Food and Drug Administration, Silver Spring, MD, United States; ^5^ Department of Chemical and Biomolecular Engineering, Johns Hopkins University, Baltimore, MD, United States; ^6^ Department of Surgery, Johns Hopkins University School of Medicine, Baltimore, MD, United States; ^7^ Department of Molecular Microbiology and Immunology, Bloomberg School of Public Health, Johns Hopkins University, Baltimore, MD, United States

**Keywords:** sialic acid, amidation, SARS-CoV-2, glycoproteomics, site occupancy, N-glycosylation, glycan shield, cell substrate

## Abstract

The severe acute respiratory syndrome coronavirus 2 (SARS-CoV-2) virus utilizes the extensively glycosylated spike (S) protein protruding from the viral envelope to bind to angiotensin-converting enzyme-related carboxypeptidase (ACE2) as its primary receptor to mediate host-cell entry. Currently, the main recombinant S protein production hosts are Chinese hamster ovary (CHO) and human embryonic kidney (HEK) cells. In this study, a recombinant S protein truncated at the transmembrane domain and engineered to express a C-terminal trimerization motif was transiently produced in CHO and HEK cell suspensions. To further evaluate the sialic acid linkages presenting on S protein, a two-step amidation process, employing dimethylamine and ammonium hydroxide reactions in a solid support system, was developed to differentially modify the sialic acid linkages on the glycans and glycopeptides from the S protein. The process also adds a charge to Asp and Glu which aids in ionization. We used MALDI-TOF and LC-MS/MS with electron-transfer/higher-energy collision dissociation (EThcD) fragmentation to determine global and site-specific N-linked glycosylation patterns. We identified 21 and 19 out of the 22 predicted N-glycosites of the SARS-CoV-2 S proteins produced in CHO and HEK, respectively. It was found that the N-glycosite at 1,158 position (N1158) and at 122, 282 and 1,158 positions (N122, N282 and N1158) were absent on S from CHO and HEK cells, respectively. The structural mapping of glycans of recombinant human S proteins reveals that CHO-Spike exhibits more complex and higher sialylation (α2,3-linked) content while HEK-Spike exhibits more high-mannose and a small amount of α2,3- and α2,6-linked sialic acids. The N74 site represents the most abundant glycosite on both spike proteins. The relatively higher amount of high-mannose abundant sites (N17, N234, N343, N616, N709, N717, N801, and N1134) on HEK-Spike suggests that glycan-shielding may differ among the two constructs. HEK-Spike can also provide different host immune system interaction profiles based on known immune system active lectins. Collectively, these data underscore the importance of characterizing the site-specific glycosylation of recombinant human spike proteins from HEK and CHO cells in order to better understand the impact of the production host on this complex and important protein used in research, diagnostics and vaccines.

## Introduction

The ongoing outbreak of severe acute respiratory syndrome coronavirus 2 (SARS-CoV-2) remains a major global pandemic affecting the lives of billions of people worldwide (Yuen et al., 2020). The pathogen of SARS-CoV-2 coronavirus causes fever and cough, shortness of breath, and hospitalization for pneumonia (Guo et al., 2020; Wiersinga et al., 2020). In view of its mortality, human-to-human and cross-species transmission ability, and virus mutation rate, there is a continuing need for effective diagnosis, efficient therapeutic treatment, and preventive means, including vaccines against this devastating disease (Wiersinga et al., 2020). As an enveloped virus, SARS-CoV-2 utilizes an extensively glycosylated spike (S) protein protruding from the viral envelope to bind to angiotensin-converting enzyme-related carboxypeptidase (ACE2) as its primary receptor (Bangaru et al., 2020; Wrapp et al., 2020). Current diagnostics, vaccine development, and neutralizing antibodies are all focused on S glycoprotein, which is the main humoral immune response target (Bangaru et al., 2020).

As a trimeric class I fusion protein, SARS-CoV-2 S has 1,273 amino acids (aa), consisting of a 13-aa signal peptide at the N-terminus, S1 subunit (14–685 aa) and membrane associated S2 subunit (686–1,273 aa) (Grant et al., 2020; Ghorbani et al., 2021). The S1 domain is responsible for binding to cell receptors, including the N-terminal domain (14–305 aa) and the receptor-binding domain (RBD, 319–541 aa); while the S2 domain mediates fusion between the virus and the cell membranes, which is compromised of the fusion peptide (FP) (788–806 aa), heptapeptide repeat sequence 1 (HR1) (912–984 aa), HR2 (1,163–1,213 aa), TM domain (1,213–1,237 aa), and cytoplasmic domain (1,237–1,273 aa) (Huang et al., 2020). The SARS-CoV-2 S binds to its receptor human ACE2 through the RBD region in its S1 subunit to initiate viral infection. Subsequently, human proteases proteolytically cleave the S protein at the S1/S2 and the S2’ sites, which allows the virus to enter for membrane fusion (Belouzard et al., 2009; Glowacka et al., 2011; Hoffmann et al., 2020). The glycan profile of envelope glycoproteins can affect the pathobiological activities and characteristics of many viruses, such as glycoprotein folding, trafficking and stability, virus release, functional activity, and immune evasion including shielding of the peptide backbone by glycans (Watanabe et al., 2019). Similar to the SARS outbreak in 2003, the SARS-CoV-2 spike utilizes glycan shielding to thwart the host immune response. Here the surface of the virus envelope is coated with host-derived glycans (Casalino et al., 2020), which hides the protein surface to avoid detection by the body fluids and cellular components of the immune system (Sommerstein et al., 2015; Pritchard et al., 2015; Watanabe et al., 2020a), thereby promoting pathogen immune evasion. Moreover, when the viral glycoprotein evolve to mask immunogenic epitopes with particularly dense host-derived glycan arrays, such as high abundance of high-mannose N-glycans, neutralizing antibodies may not always recognize the underlying viral protein surface (Grant et al., 2020). Recently, a research group discovered that SARS-COV-2 spike glycoprotein glycans shield approximately 40% of the protein surface, although the glycans only account for 17% of the total molecular weight of the S trimer when expressed from human embryonic kidney cells (Grant et al., 2020; Yang et al., 2020). Molecular dynamic simulation of the interaction of spike and ACE2 proteins revealed the role of spike glycans in sterically masking polypeptide epitopes and directly participates in spike-ACE2 interactions (Zhao et al., 2020). Thus, understanding the glycosylation of recombinant S protein is very important for studying virus biology and immune response, and also help to provide information for the application of recombinant spike glycoprotein in diagnosis and vaccines.

As one of the most complex post-translational modifications found on secreted proteins and membrane-bound proteins, glycosylation can affect numerous physiological and pathological cell functions. Compared with other post-translational processes events, glycosylation is unique in its structural heterogeneity. The glycan pattern can vary greatly among individual glycosites of a given protein, different organisms, and different production hosts. Therefore, the characterization of glycans has presented a special analytical challenge due to the extraordinary heterogeneity and complexity caused by the number of glycan processing enzymes residing in the ER and Golgi apparatus. However, the advent of powerful mass spectrometry tools and related analysis methods has revolutionized our ability to identify and characterize protein glycosylation (Nwosu et al., 2011; Han and Costello, 2013; Sun et al., 2016). After the outbreak of the pandemic, multiple research groups reported glycomic and glycoproteomic analysis of the SARS-CoV-2 spike protein (De Leoz et al., 2020; Shajahan et al., 2020; Watanabe et al., 2020b; Sanda et al., 2021). However, these studies have not yet compared the production of spike proteins in multiple mammalian production hosts or characterized their sialic acid linkages. Given that the two most important potential commercial recombinant protein production hosts are Chinese hamster ovary (CHO) and human embryonic kidney (HEK) cells, comparing the glycosylation profiles of recombinant spikes produced by these two organisms is useful for understanding what host-specific differences in glycosylation are and the potential relevance of these differences. Indeed, the differences in glycosylation between hamster ovary and human kidney cells may result in distinct glycan profiles of the same recombinant protein. For example, a comparison of 12 glycoproteins expressed from HEK and CHO suspension cell cultures revealed distinctive glycan structures, and CHO cells tend to express higher levels of sialic acid (Croset et al., 2012). In addition, CHO cells present exclusively α2,3-sialylation and trace amounts of Neu5Gc (Wang et al., 2015; Yehuda and Padler-Karavani, 2020). In contrast, HEK cells can simultaneously exhibit α2,3- or α2,6-linked sialylation. Sialic acids (Neu5Ac) are typically located at the terminus of oligosaccharides on glycoproteins, and these residues are mainly linked to the galactose residue by α2,3- or α2,6-linkages in humans (Varki et al., 2015; Zhou et al., 2020). In fact, differences in specific linkages can affect the function of viral protein such as the entry protein hemagglutinin of influenza A virus with an α2,6-linked sialic acid binding preference (Leung et al., 2012; Yang et al., 2017).

**FIGURE 1:**
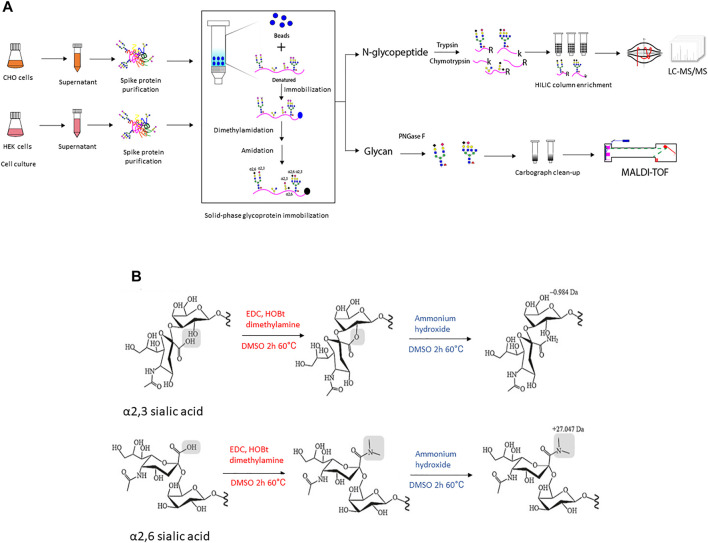
**(A)** The illustration of experimental workflow in this project. Recombinant SARS-CoV-2 spike protein was transiently expressed in CHO and HEK cells in serum-free media, respectively. The conditioned supernatant was harvested and spike protein with a 6xhis tag at C-terminal was purified through Ni-NTA column and the protein purity was verified using SDS-PAGE followed by Coomassie blue staining (Supplementary Figure S2). Then equal amounts of purified recombinant spike protein were denatured and conjugated to an aldehyde-activated bead-based solid phase for N-glycan/N-glycopeptide analysis. The conjugated glycopeptides were subjected to reductive amination, lactonization and dimethylamidation for individual α2,3- and α2,6-linked sialic acids labeling. Subsequently, for N-glycopeptide analysis, trypsin and chymotrypsin digestion was performed and the flow-through digested N-glycopeptides were enriched using the HILIC column. The enriched N-glycopeptides were analyzed using LC-MS/MS mass spectrometry. Meanwhile, for N-glycan analyses of recombinant spike proteins from each cell line, PNGase F digested was applied to release N-glycans, followed with Carbograph column for N-glycan clean-up. Then N-glycans were analyzed using MALDI-TOF mass spectrometry. **(B)** The schematic illustration of two-step reactions in this project. The first step–dimethylamine treatment under EDC + HOBt condition, α2,3-sialic acid forms a lactone to the neighbor galactose, while α2,6-sialic acid forms a stable dimethyl amid structure. The second step-ammonium hydroxide treatment, α2,3-sialic acid further formed a stable amidation structure, while α2,6-sialic acid formed dimethyl amide remains stable in ammonium hydroxide treatment.

Therefore, in this study, recombinant full-size spike protein was transiently produced in suspension propagated CHO and HEK hosts. The resulting glycan and glycopeptide profiles were characterized in order to explore the different capabilities and characteristics of recombinant spike glycosylation. To further evaluate the presence of sialic acid linkages on the S glycans, a solid-phase method was used for two-step derivatization with dimethylamine and ammonium hydroxide to differentially modify the sialic acid linkages on glycans and glycopeptides from spike proteins secreted from the two hosts. Figure 1 shows the schematic workflow of the glycan and glycoproteomic analytical process. We provided global and site-specific analysis of N-linked glycosylation on soluble full-size SARS-CoV-2 spike using MALDI-TOF and LC-MS/MS with electron-transfer/higher-energy collision dissociation (EThcD) fragmentation. It revealed extensive heterogeneity ranging from high-mannose type to complex type glycosylation profiles, and has detailed sialic acid linkage information. The structural mapping of glycans of recombinant human spike proteins shows that CHO-Spike is more complex and contains higher sialylation (α2,3-linked), while HEK-Spike has more high-mannose and a small amount of α2,3- and α2,6-linked sialylation. Some high-mannose abundant sites on HEK-Spike may indicate a comparatively different glycan shielding presentation and viral evasion ability compared with CHO-Spike, which interacts with high mannose specific lectins such as DC-SIGN and lung surfactant SP-D (Van Breedam et al., 2014). Overall, these data underscore the importance of characterizing glycosylation of recombinant human spike protein from HEK and CHO cells in order to better understand the impact of the production host on S protein used in research, diagnostics and vaccines.

## Materials and Methods

### Cell Culture, Transfection and Protein Purification

The mammalian expression vector pCAGGS plasmid containing the stable soluble spike protein sequence was acquired from Dr. Andrew Pekosz of Bloomberg School of Public Health at Johns Hopkins University through the generosity of Dr. Florian Krammer of the Icahn School of Medicine at Mt. Sinai (Amanat et al., 2020; Stadlbauer et al., 2020). Substitutions at the furin cleavage sites and lysine (K) at 986 position and valine (V) at 987 position were replaced with prolines (P) in order to stabilize the quaternary structure for mammalian glycosylation processing (Watanabe et al., 2020b). This recombinant S protein was truncated at the transmembrane domain and engineered to express a C-terminal trimerization motif. A sequence comparison with the SARS-CoV-2 Wuhan strain is shown in Supplementary Figure S1. The CHOZN GS−/− cell line was acquired from MilliporeSigma. Suspension HEK 293 cells were acquired from the American Type Culture Collection (ATCC). For CHO cells, cell culture was maintained in EX-CELL CD CHO Fusion medium (MilliporeSigma) supplemented with 6 mM glutamine in 250 ml shake flasks with a working volume of 100 ml at 37°C and 8% CO_2_. Transfection and transient expression of spike protein were performed using reagents and media from CHOgro® Expression System (Mirus Bio) following the corresponding protocol. The protein was harvested from 300 ml spent media 4 days after transfection. The protein was purified *via* the Ni-NTA purification approach (Spriestersbach et al., 2015). For HEK cells, cell culture was maintained in FreeStyle™ F17 Expression Medium (Thermo Fisher) supplemented with 6 mM glutamine in 250 ml shake flasks with a working volume of 100 ml at 37°C and 8% CO_2_. Transfection was performed *via* a polyethyleneimine (PEI)-based approach (Longo et al., 2013). Specifically, 1 mg/ml of PEI in water was prepared and added to Opti-Pro SFM (Thermo Fisher) containing plasmid DNA in a 3.5:1 PEI to DNA ratio (w/w) to form the DNA-PEI complex. Spent media was collected 4 days after transfection and the protein was purified *via* the Ni-NTA purification approach. The purified spike protein with a 6xHis tag at the C terminal was evaluated using SDS-PAGE with Coomassie blue staining to check any impurities, as shown in Supplementary Figure S2. The protein concentration was measured by bicinchoninic acid assay (BCA assay).

### Solid-phase Protein Conjugates and Modifications

Purified S protein, 400 μg, was diluted in 500 µL solution containing 1x binding buffer and 1x denaturing buffer (New England BioLabs, B1704S) and denatured at 95°C for 5 min. 1x binding buffer was prepared by dissolving 294 mg sodium citrate and 53 mg sodium carbonate in 10 ml HPLC water. All chemicals were purchased from Sigma-Aldrich unless otherwise noted. Meanwhile, Aminolink resin (Fisher Scientific) was washed by 500 µL 1x binding buffer twice. Samples were then denatured, added to the pre-conditioned resin in a snap-cap spin column (SCSC, Fisher Scientific), and incubated overnight at room temperature. The 50 mM NaCNBH_3_ reduction solution was added to the sample for another 4 h incubation. The resin was then washed with 1x PBS twice and further incubated with 50 mM NaCNBH_3_ in PBS for another 4 h to block any active sites.

For sialic acid modification, samples were first treated with 200 µl 0.25 mol/L dimethylamine, 0.25 mol/L 1-ethyl-3-(3-dimethylamino)propyl)carbodiimide (EDC) and 0.25 mol/L 1-hydroxybenzotriazole (HOBt) in DMSO solution at 60°C for 2 h. EDC and HBOt were used as a carboxylic acid activator and a catalyst individually. Next, an equal volume of ammonium hydroxide (pH10) was added into the samples with another 2 h incubation at 60°C. Samples were then washed sequentially with 10% formic acid, 10% acetonitrile, 1 M NaCl and H_2_O, each for three times. Finally, the sialic acid-modified protein conjugates were subjected to glycan and glycopeptide analysis individually.

### N-Glycan Analysis

After solid-phase protein conjugates and modifications, 3ul of PNGase F (500,000 U/ml, New England BioLabs, Ipswich, MA) in NEB Glycobuffer 2 was added to the bead mixture and incubated overnight at 37°C. The extracted glycans were subjected to Carbocolumn N-glycan clean-up and stored at 4°C for MALDI-ToF analysis. The purified glycan was analyzed using a Bruker AutoFlex Speed MALDI-ToF/ToF spectrometer in the reflective-positive ion mode. The MALDI-ToF MS parameters were set as following: mass range 800–6,000 Da, laser 70% and 8,000 summed shots per sample. Search for predicted glycan structures-based compositions was performed using GlycoWorkBench software. The α2,3-linked sialic acids formed a stable amidation structure with a mass shift of −0.984 Da, while the α2,6-linked sialic acids formed a stable dimethyl amidation structure in ammonium hydroxide with a mass shift of +27.047 Da.

### On-Bead N-Glycopeptide Digestion and HILIC Enrichment

After protein conjugation and modification, the resin linked protein was incubated in 12 mM dithiothreitol (DTT) in 1M NH_4_HCO_3_ and 8 M urea at 37°C for 1 h. Iodoacetamide was then added to a final concentration of 16 mM (1 h at room temperature in the dark) to alkylate the protein. After alkylation, samples were washed by 1M NaCl, HPLC water and 25 mM NH_4_HCO_3_ sequentially (twice). For fetuin sample, sequence-grade trypsin digestion (protein: enzyme = 50:1, w/w) in 50 mM NH_4_HCO_3_ was performed at 37°C overnight. For spike samples, sequence-grade trypsin and chymotrypsin (as a cocktail) digestion (protein: enzyme = 50:1, w/w) in 50 mM NH_4_HCO_3_ was performed at 37 °C overnight. The sample digest was then eluted in 80% ACN and subjected to HILIC N-glycopeptide enrichment. HILIC SPE chromatography was prepared as follows: add empty SPE (solid-phase extraction) frits to Grace Alltech extract-clean empty reservoir (1.5 ml; Fisher Scientific), load 500 ul TSKgel Amide-80 slurry in 50% ethanol (Sigma-Aldrich), and cap resin using empty SPE frits.

HILIC column enrichment was performed as follows: pre-condition HILIC column using 0.1% TFA and 60% ACN/0.1% TFA (1 ml, three times), load samples in 80% ACN/0.1% TFA (reload flow-through once), wash column by 80% ACN/0.1% TFA (1ml, twice), elute samples by 60% ACN/0.1% TFA, 40% ACN/0.1% TFA, and 0.1% TFA, and pool samples. The eluates were dried by rotary evaporation in a Speed-Vac instrument (Thermo Fisher) and re-suspended in 0.2% FA.

### Nano LC-MS/MS Analysis

The dried peptide sample was resuspended in 0.2% formic acid (FA) and measured peptide concentration using A280 Nanodrop. Then, 1 µg of resuspended glycopeptide in 4 µL FA buffer were analyzed by LC-MS/MS using a Thermo Orbitrap Fusion Lumos mass spectrometer (Thermo Fisher Scientific) for each run. The analytical method was adapted from a previous publication (Wang et al., 2021). Briefly, glycopeptides were first loaded and de-salted with a trap column (Thermo Fisher PepMap™, C18, 3 μm, 100 Å, 75 μm × 2 cm) at 5 μL/min with 100% Solvent A (0.1% formic acid in HPLC water) for 5 min. Then, glycopeptides were separated by an Accalaim PepMap™ 100 nano column (3 μm, 100 Å, 75 μm × 250 mm) using a linear gradient of 2.5–37.5% solvent B (80% ACN, 0.1% formic acid) over 85 min, with a wash at 90% B for 5 min. Data-dependent analysis (DDA) was carried out with a duty cycle of 2 s. Precursor masses were detected in the Orbitrap at resolution (R) = 120,000 (at m/z 200) with internal calibration (Easy IC). Stepped HCD spectra (HCD energy at 15, 25, and 35%) were acquired for precursors with charges between two and eight and intensities over 5.0 × 10^4^ at R = 30,000. Dynamic exclusion was set at 20 s. If at least one of the three common glycan oxonium fragment ions (m/z 138.0545, 204.0867, and 366.1396 Da) was observed within 15 ppm mass accuracy, EThcD acquisition were performed in the orbitrap at R = 30,000. The electron-transfer dissociation (ETD) reagent target was 2.0 × 10^5^, with supplemental collision energy at 15%. The ETD reaction time was dependent on the precursor charge state: 125 ms (ETD reaction time) for charge 2, 100 ms for 3, 75 ms for 4, and 50 ms for ≥5 (Wang et al., 2021).

### Data Processing and Bioinformatics Analysis

Peptide identification was performed using Byonic version 4.0 and glycopeptide quantification and characterization was performed by Byologic version 4.0 (Protein Metrics Inc., San Carlos, CA). The Byonic software parameters are listed in Supplemental Table S1. The precursor mass tolerance was 10 ppm, and the fragment mass tolerance was 15 ppm. The manual score cutoff was 50, the PEP2D score was less than 0.5, and the protein false discovery rate (FDR) was 1%. To accommodate for amide-modified Neu5NAc and Neu5Gc masses, as incurred with use of dimethylamine and ammonium hydroxide, we built a mammalian N-glycan database to include these modifications. Dimethylamine can also modify the carboxyl groups on Aspartic acid (D), Glutamic acid (E) and the C-terminal of peptides. We noted this derivatization as a fixed modification in the Byonic search. We performed data searches using dimethylamination only (Option 1) and both dimethylamination and amidation (Option 2) on the same LC-MS/MS experimental dataset using modified fetuin. The search result from Option two showed that D and E are principally modified by dimethylamination and only a trace amount was modified by amidation. According to these results, we use Option one in Byonic as the modification of D, E, and C-terminal of glycopeptides. Using Byonic search results as inputs, Byologic can compute extracted ion chromatograms (XICs) and relative abundances using a label-free quantification approach, a default setting in the Byologic software. The relative abundance of individual glycoforms on specific glycopeptides was calculated by XIC intensities over all charge states. The results of Byologic analysis were further processed and organized using the RStudio program software (Team, 2020). The mass spectrometry glycoproteomics data have been deposited to the ProteomeXchange Consortium *via* the PRIDE (Vizcaíno et al., 2016) partner repository with the dataset identifier PXD027536 (Deutsch et al., 2017) at http://www.ebi.ac.uk/pride.

## Results and Discussion

### Evaluation of Differential Sialic Acid Linkage Labeling of Fetuin Glycopeptides

Several derivatization methods have recently been developed to effectively stabilize sialic acid residues and distinguish the sialyl linkage isomers (Holst et al., 2016; Yang et al., 2018). For example, the application of dimethylamine (NH(CH_3_)_2_) with carboxylic acid activator EDC and the catalyst HOBt in a DMSO solution can result in lactonization and dimethylamidation for individual α2,3- and α2,6-linked sialic acids, respectively (Figure 1B) (de Haan et al., 2015). Furthermore, the two-step derivatization by dimethylamine and ammonium hydroxide improved this dimethylamine derivation stability by converting α2,3-sialic acid lactonization into amidation in ammonium hydroxide solution while α2,6-sialic acid dimethylamidation remains stable (Holst et al., 2016; Zhou et al., 2019). This two-step method modified the sialic acid and made it easier to detect different linkages of sialic acids on glycans using MALDI-TOF MS (Zhou et al., 2019). In the current study, we applied this two-step sialic acid derivatization method to glycopeptide analysis using a solid support system. The specific sialic acid linkages on N-glycans of fetuin from fetal bovine serum were first evaluated as a model protein using a solid support that facilitates glycomic and glycoproteomic measurements. The differential sialic acid linkage labeling method used to generate the fetuin N-glycan profile is shown in Supplementary Figure S3. The resulting detailed released N-glycan quantification is listed in Supplementary Table S2, which is consistent with the fetuin sialylated N-glycan profiles reported in the literature (Nwosu et al., 2011; Yang and Zhang, 2014), and in agreement with reports that differentiate between terminal sialic acid linkage types identified by alternative labeling strategies (Yang et al., 2017) and high field NMR (Green et al., 1988; Hayase et al., 1992).

Next, this two-step differential sialic acid labeling method was performed on fetuin glycopeptides using the solid support system. To determine whether sialic acids on intact glycopeptides were successfully derivatized by dimethylamine/NH₄OH, we inspected the fragment ions of tandem mass spectra. Representative glycopeptides KLC [+57]PD [+27.047]C [+57]PLLAPLN [2886.0791]DSR and VVHAVE [+27.0470]VALATFNAESN [3279.2423]GSYLQLVEISR were observed as shown in Supplementary Figure S4 (upper lanes), which contain oxonium ions bearing mass shifts with both dimethylamine and NH₄OH modified Neu5Ac ions. Other oxonium ions, C6H8NO2, C7H8NO2, HexNAc-H_2_O, HexNAc, HexNAcHex and HexNAxHex (2) were also observed. These two glycopeptides have only one N-glycan modified by dimethylamine and NH₄OH. The fragment peaks are present at 308.289 Da for NeuAc-H_2_O + NH_3_ (α2,3-linked) and 336.32 Da for NeuAc-H_2_O + NH(CH_3_)_2_ (α2,6-linked) as shown in Supplementary Figure S4. These results verified that sialic acids are successfully modified by dimethylamidation and amidation. Moreover, compared to the native fetuin glycopeptide (Supplementary Figure S4 lower panels) under the same experimental condition, we observed that more oxonium ions were detected and at higher intensity when dimethylamine and NH₄OH modified Neu5Ac for the labeled fetuin glycopeptides compared to unmodified Neu5Ac of native fetuin glycopeptides. Additionally, we also noticed that the labeled glycopeptide has a higher mass charge state, which may be due to the dimethylamidation of the carboxyl groups of Aspartic acid (D), Glutamic acid (E) and the C-terminal of peptides, where primary amines form -NH_3_^+^ ions at a lower pH (i.e., 0.1% TFA).

**FIGURE 2:**
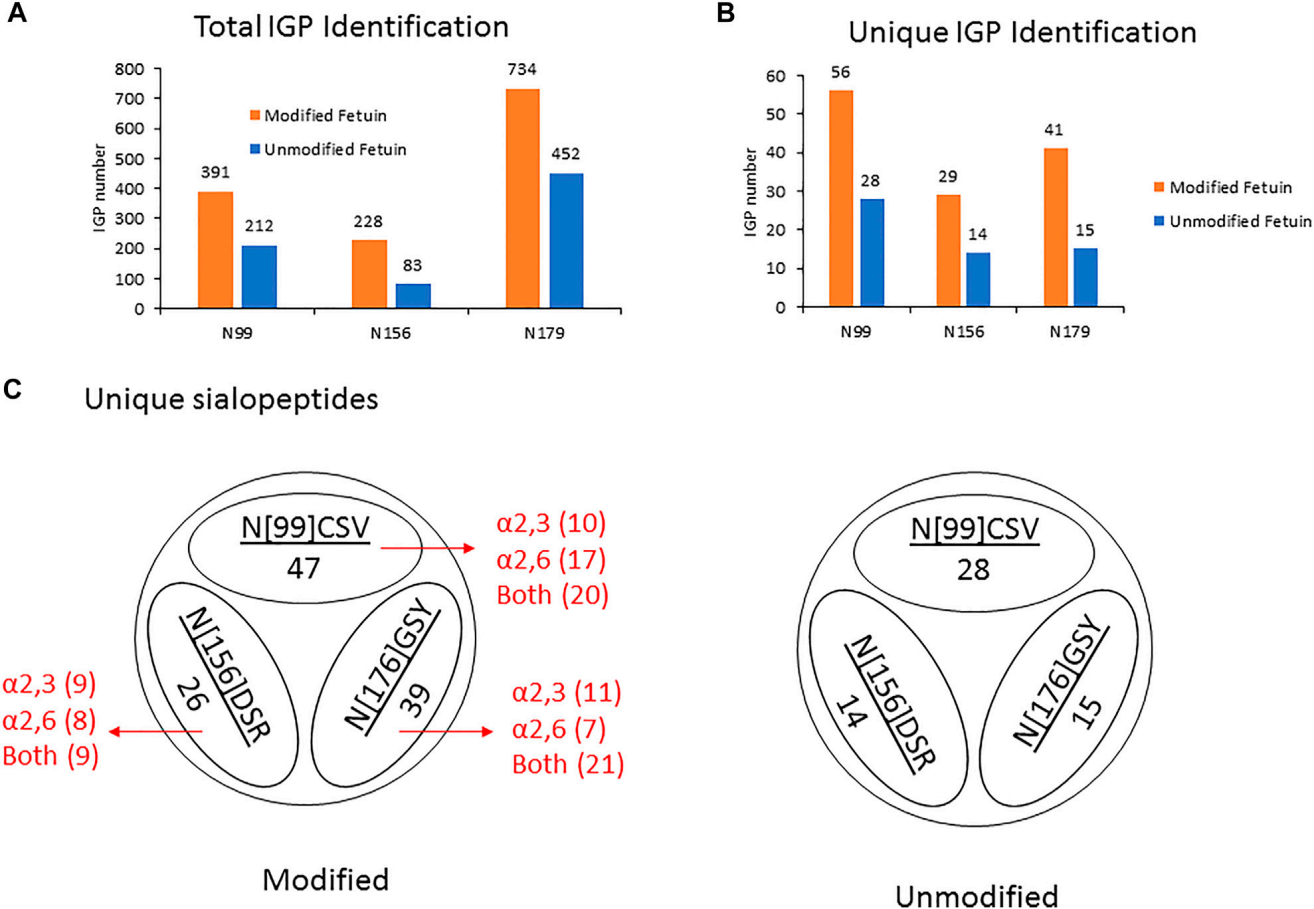
**(A,B)** The site-specific summary of total IGP **(A)** and unique IGP **(B)** of modified and unmodified fetuin glycopeptides **(C)** the site-specific unique sialopeptide analysis modified and unmodified fetuin glycopeptides.

Thus, by using 3 µg of the modified and unmodified intact glycopeptides, performed with three analytic repeats of fetuin, the total and unique intact glycopeptides (IGP) identified for the labeled samples were 1,353 and 126, compared to 747 and 57 in native sample separately. The site-specific total and unique IGP distribution are presented in Figures 2A,B, in which the sialic acid labeled glycopeptide showed more IGP identified compared to native glycopeptides at each N-glycan site. As mentioned above, derivatization can also occur on the carboxylic acids of D, E, or the C-terminus. Supplementary Table S3 lists the relative abundance of glycopeptides containing D and/or E residues modified by dimethylamine in the analysis of fetuin. The glycopeptides at N99 and N179 sites contain a higher number of D and E residues, and the signal strength is higher. On the other hand, the glycopeptide at N156 site have fewer D and E residues, resulting in lower charge state and signal intensity in EThcD fragmentation. Moreover, the site-specific unique sialopeptide analysis (Figure 2C) identified more unique sialopeptides in modified glycopeptides versus unmodified fetuin owing to the linkage specific mass shifts imparted by the labeling strategy. Furthermore, the identified linkage specific unique sialopeptides account for the majority of the identified unique IGPs (Figures 2B,C), indicating that fetuin is a highly sialylated glycoprotein. Over 75 unique N-glycans were identified in modified fetuin and 24 unique N-glycans without sialylation linkage information were identified for native fetuin with the detailed site-specific N-glycan profile tabulated in Supplementary Table S4.

### Characterization of Recombinant SARS-CoV-2 Spike Proteins Secreted From CHO or HEK Cell Culture

**FIGURE 3:**
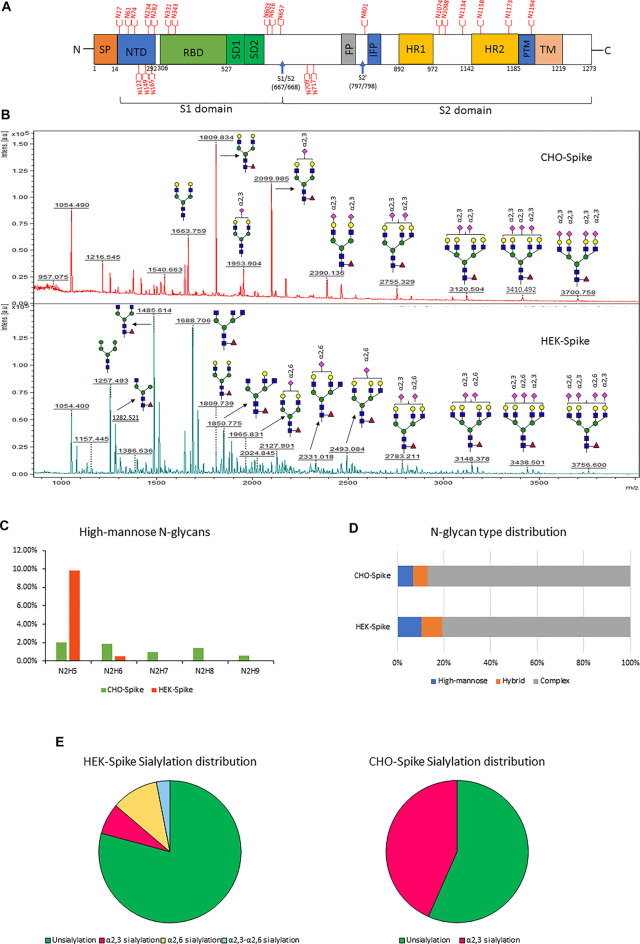
**(A)** Schematic diagram of the SARS-CoV-2 Spike protein. Blue arrows indicate the S1–S2 and S′ proteolytic cleavage sites at residues R667 and R797, respectively. 22 N-glycan sites are labeled in red. **(B)** the glycomic analysis of released N-glycans of recombinant full-size spike protein from CHO **(upper lane)** and HEK **(lower lane)** with sialic acids modified by dimethylamidation and amidation, depending on their linkages. **(C–F)** the summaries of high-mannose types **(C)**, N-glycan type distribution **(D)** and sialylation linkage distribution **(E)** identified from released N-glycans of CHO-Spike and HEK-Spike.

After evaluating the dimethylamine-NH₄OH sialic acid linkage labeling method on fetuin, we next applied this method for the glycomic and glycoproteomic analysis of full-size recombinant SARS-CoV-2 spike protein expressed by either CHO-GS or HEK293 suspension cells. Currently, CHO and HEK cells are the two predominant production platforms for recombinant soluble S protein. Given that CHO and HEK have distinctive glycosylation processing pathways (Croset et al., 2012), the resulting soluble S proteins may produce different N-glycan profiles. Indeed, due to the lack of α2,6-sialyltransferase expression, CHO cells usually only have α2,3-sialylated glycans (Hossler et al., 2009), while HEK cells typically have both α2,3- and α2,6-linked sialic acids. CHO cells may also produce small amounts of Neu5Gc, which can be recognized as a foreign epitope and cause immunogenicity in humans (Borys et al., 2010). A schematic illustration of the different regions of SARS-CoV-2 S protein is presented in Figure 3A. According to literature, the SARS-CoV-2 S protein is heavily glycosylated and has 22 N-glycosites (Watanabe et al., 2020b) as designated in Figure 3A. Glycan shielding provides a thick sugar-coated barrier against neutralizing antibody recognition. Consequently, global and site-specific deciphering of N-glycosylation of SARS-CoV-2 S protein from different mammalian expression hosts will help characterize recombinant spike proteins, diagnose COVID-19, and develop protein vaccine.

**FIGURE 4:**
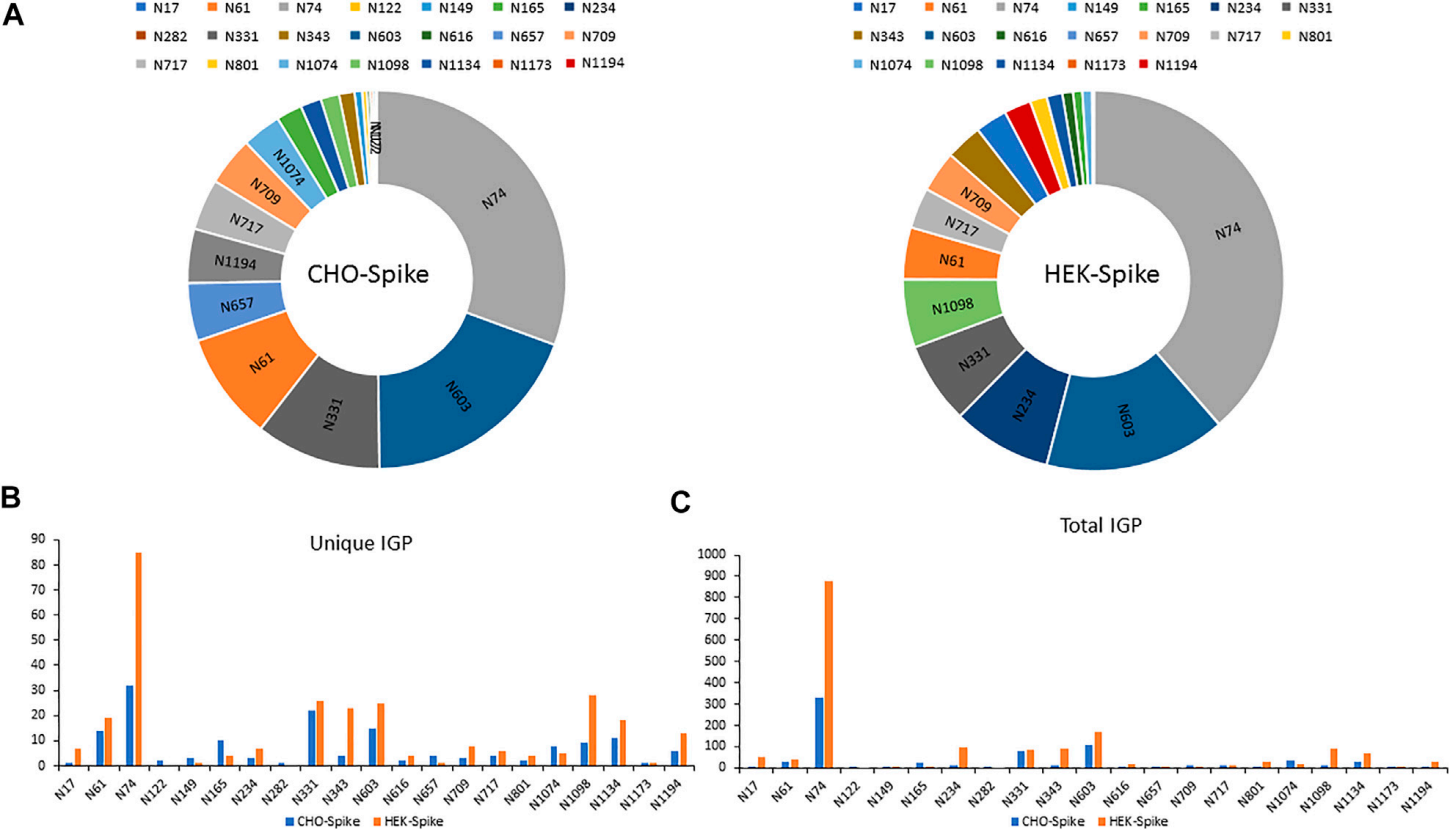
**(A)** The identified N-sites and their relative abundance of glycopeptides from CHO-Spike and HEK-Spike proteins. The site-specific summary of unique IGP **(B)** and total IGP **(C)** of glycopeptides identified from CHO-Spike and HEK-Spike proteins.

The released N-glycan MS profile from three glycopeptide analytical replicates of the spike proteins from CHO and HEK hosts are shown in Figure 3B. The determined glycan compositions and percentage levels are listed in Tables 1, 2. In total, 44 and 56 N-glycan types were identified in the spike proteins from CHO and HEK cells, respectively. The distribution of high-mannose and N-glycan types is presented in Figures 3C,D. CHO-Spike mainly displays unsialylated and α2,3-monosialyated complex N-glycans, while HEK-Spike shows more high-mannose glycans, mainly Man_5_GlcNAc_2_, with less distribution between large and small MW glycans compared with CHO-Spike. HEK-Spike also mainly contains unsialylated bi- and tri-antennary N-glycans and a small amount of various α2,3- and α2,6-sialylated complex types. In accordance with our result in Figures 3C,D, another group also found that high oligomannose and highly fucosylated bi- and tri-antennary N-glycans have low sialylation content on the spikes of HEK cells. However, here we used solid-phase sialic acid linkage method to improve the content of the information (Zhao et al., 2020). Indeed, different expression systems and protein constructs may present different glycan patterns. The α2,3- and α2,6-sialylation distribution summary from glycomic analysis (Figure 3E) also reveals that HEK-Spike contained 7 and 11% exclusive α2,3- and α2,6-sialylation individually and 3% mixed sialylation, while CHO-Spike only bears 43% α2,3-sialylation. Meanwhile, the site-specific N-glycosylation analysis of S protein was performed under the co-digestion of trypsin and chymotrypsin. The representative MS/MS spectra are presented in Supplementary Figure S5. We identified the glycan compositions at 21 and 19 out of the 22 predicted N-glycosylation sites of SARS-CoV-2 S proteins in CHO and HEK, respectively. The determined N-glycosites and their relative abundance based on LC-MS signal intensity (percentage of total glycopeptide LC-MS intensity) are displayed in Figure 4A. We quantified the relative intensity of glycans at each site by comparing the area under the curve of each glycopeptide peak on the LC-MS chromatogram. Interestingly, in this study, the CHO derived spike N-glycosite at 1,158 position (N1158) was unoccupied while the N-glycosites at 122, 282 and 1,158 positions (N122, N282 and N1158) were found unoccupied in the HEK derived spike. Another spike protein glycopeptide mapping analysis identified 17 out of 22 predicted N-sites from SARS-CoV-2 to be occupied when S1 and S2 protein fragments were expressed separately from HEK cells using a stepped HCD (higher-energy collisional dissociation) triggered CID (collision-induced dissociation) program (Shajahan et al., 2020) with 17, 603, 1,134, 1,158 and 1,173 positions unoccupied. We identified 19 out of 22 N-sites from full-size spike from HEK cells to be occupied. The identified differences may be attributed to the dissimilar LC-MS/MS fragmentation program applied, the sialic acids status as labeled or not, the size of proteins analyzed (full-size spike or individual S1 and S2 proteins), and the polypeptide processing in ER and *Golgi* apparatus. Alternatively, the N74 site represents the most abundantly occupied N-site of Spike from both CHO and HEK cells as displayed in Figures 4B,C for site-specific total and unique IGP numbers summed from three analytical repeats. Furthermore, the HEK-Spike protein analysis identified more unique IGPs at N74 site, indicating the site may be more heavily glycosylated on HEK-Spike than CHO-Spike.

**TABLE 1:** Released N-glycans identified from CHO-Spike protein.

Experimental Mass [M + Na]+	Theoretical Mass [M + Na]+	Delta Mass	Composition	Relative percentage
1136.610	1136.40	0.209	N3H3	0.56%
1257.570	1257.43	0.142	N2H5	2.04%
1282.621	1282.46	0.161	N3H3F1	0.58%
1298.591	1298.45	0.137	N3H4	0.77%
1419.630	1419.48	0.149	N2H6	1.86%
1444.633	1444.51	0.121	N3H4F1	0.79%
1460.649	1460.51	0.142	N3H5	0.71%
1485.683	1485.54	0.145	N4H3F1	1.22%
1501.680	1501.53	0.146	N4H4	1.36%
1581.713	1581.53	0.179	N2H7	0.93%
1588.703	1588.57	0.137	S(23)1-N3H4	0.37%
1647.757	1647.59	0.165	N4H4F1	4.00%
1663.759	1663.59	0.172	N4H5	7.09%
1743.668	1743.59	0.082	N2H8	1.41%
1750.754	1750.62	0.135	S(23)1-N3H5	0.62%
1791.775	1791.65	0.129	S(23)1-N4H4	0.85%
1809.834	1809.64	0.190	N4H5F1	22.89%
1825.837	1825.64	0.198	N4H6	0.77%
1850.848	1850.67	0.177	N5H4F1	0.47%
1905.804	1905.64	0.165	N2H9	0.56%
1930.790	1930.67	0.120	N3H7F1	1.78%
1937.910	1937.70	0.207	S(23)1-N4H4F1	2.21%
1953.904	1953.70	0.206	S(23)1-N4H5	4.43%
2012.927	2012.72	0.203	N5H5F1	1.38%
2028.911	2028.72	0.193	N5H6	0.88%
2099.985	2099.76	0.229	S(23)1-N4H5F1	19.53%
2115.973	2115.75	0.222	S(23)1-N4H6	0.79%
2175.009	2174.78	0.232	N5H6F1	3.39%
2244.063	2243.81	0.253	S(23)2-N4H5	0.54%
2303.057	2302.84	0.222	S(23)1-N5H5F1	0.69%
2319.111	2318.83	0.281	S(23)1-N5H6	0.65%
2378.122	2377.86	0.266	N6H6F1	0.37%
2390.136	2389.87	0.268	S(23)2-N4H5F1	3.51%
2465.166	2464.89	0.278	S(23)1-N5H6F1	2.60%
2540.199	2539.91	0.290	N6H7F1	0.79%
2593.117	2592.95	0.170	S(23)2-N5H5F1	0.27%
2609.265	2608.94	0.323	S(23)2-N5H6	0.32%
2668.270	2667.97	0.303	S(23)1-N6H6F1	0.22%
2755.329	2755.00	0.329	S(23)2-N5H6F1	2.17%
2830.358	2830.02	0.338	S(23)1-N6H7F1	0.87%
3045.463	3045.11	0.352	S(23)3-N5H6F1	0.72%
3120.504	3120.13	0.372	S(23)2-N6H7F1	0.97%
3410.492	3410.24	0.249	S(23)3-N6H7F1	0.64%
3700.758	3700.35	0.403	S(23)4-N6H7F1	0.42%

**TABLE 2:** Released N-glycans identified from HEK-Spike protein.

Experimental Mass [M + Na]+	Theoretical Mass [M + Na]+	Delta Mass	Composition	Relative percentage
1136.451	1136.402	0.050	N3H3	1.40%
1257.493	1257.428	0.065	N2H5	9.80%
1282.521	1282.459	0.062	N3H3F1	5.18%
1298.492	1298.454	0.037	N3H4	0.56%
1339.535	1339.481	0.054	N4H3	0.88%
1419.536	1419.481	0.056	N2H6	0.54%
1444.576	1444.512	0.064	N3H4F1	1.24%
1485.614	1485.539	0.075	N4H3F1	18.34%
1501.581	1501.534	0.047	N4H4	0.70%
1606.617	1606.565	0.052	N3H5F1	0.45%
1647.670	1647.592	0.078	N4H4F1	4.87%
1688.706	1688.618	0.088	N5H3F1	16.54%
1793.718	1793.65	0.068	N4H4F2	0.77%
1809.739	1809.644	0.094	N4H5F1	2.36%
1834.773	1834.676	0.097	N5H3F2	0.72%
1850.775	1850.671	0.103	N5H4F1	4.73%
1891.802	1891.698	0.104	N6H3F1	3.65%
1937.811	1937.703	0.108	S(23)1-N4H4F1	0.92%
1955.817	1955.702	0.115	N4H5F2	0.61%
1965.831	1965.734	0.097	S(26)1-N4H4F1	1.20%
1996.848	1996.729	0.119	N5H4F2	1.18%
2012.837	2012.724	0.113	N5H5F1	1.44%
2037.871	2037.756	0.115	N6H3F2	0.47%
2053.867	2053.75	0.117	N6H4F1	1.14%
2099.891	2099.756	0.136	S(23)1-N4H5F1	1.26%
2127.901	2127.787	0.114	S(26)1-N4H5F1	2.41%
2140.901	2140.782	0.118	S(23)1-N5H4F1	1.03%
2168.922	2168.813	0.109	S(26)1-N5H4F1	1.54%
2174.914	2174.777	0.137	N5H6F1	0.99%
2215.949	2215.803	0.145	N6H5F1	0.61%
2243.935	2243.809	0.126	S(23)2-N4H5	0.37%
2303.021	2302.835	0.186	S(23)1-N5H5F1	0.85%
2331.018	2330.866	0.152	S(26)1-N5H5F1	0.87%
2372.004	2371.893	0.111	S(26)1-N6H4F1	0.44%
2418.039	2417.898	0.141	S(23)1S(26)1-N4H5F1	0.39%
2446.047	2445.929	0.118	S(26)2-N4H5F1	0.35%
2465.069	2464.888	0.181	S(23)1-N5H6F1	0.91%
2493.084	2492.919	0.165	S(26)1-N5H6F1	1.51%
2506.124	2505.915	0.209	S(23)1-N6H5F1	0.39%
2534.118	2533.946	0.173	S(26)1-N6H5F1	0.60%
2668.099	2667.967	0.132	S(23)1-N6H6F1	0.29%
2755.228	2754.999	0.229	S(23)2-N5H6F1	0.46%
2783.211	2783.03	0.181	S(26)1S(23)1-N5H6F1	0.84%
2811.223	2811.061	0.161	S(26)2-N5H6F1	0.60%
2830.265	2830.02	0.245	S(23)1-N6H7F1	0.30%
2852.277	2852.088	0.189	S(26)2-N6H5F1	0.20%
2858.258	2858.051	0.207	S(26)1-N6H7F1	0.52%
2986.321	2986.11	0.211	S(23)1S(26)1-N6H6F1	0.22%
3101.333	3101.173	0.160	S(23)1S(26)2-N5H6F1	0.24%
3120.382	3120.132	0.250	S(23)2-N6H7F1	0.28%
3148.378	3148.163	0.216	S(26)1S(23)1-N6H7F1	0.59%
3176.373	3176.194	0.180	S(26)2-N6H7F1	0.47%
3438.501	3438.274	0.227	S(26)1S(23)2-N6H7F1	0.30%
3466.510	3466.305	0.205	S(26)2S(23)1-N6H7F1	0.27%
3756.600	3756.416	0.184	S(26)2S(23)2-N6H7F1	0.13%
3784.596	3784.447	0.148	S(26)3S(23)1-N6H7F1	0.09%

**FIGURE 5:**
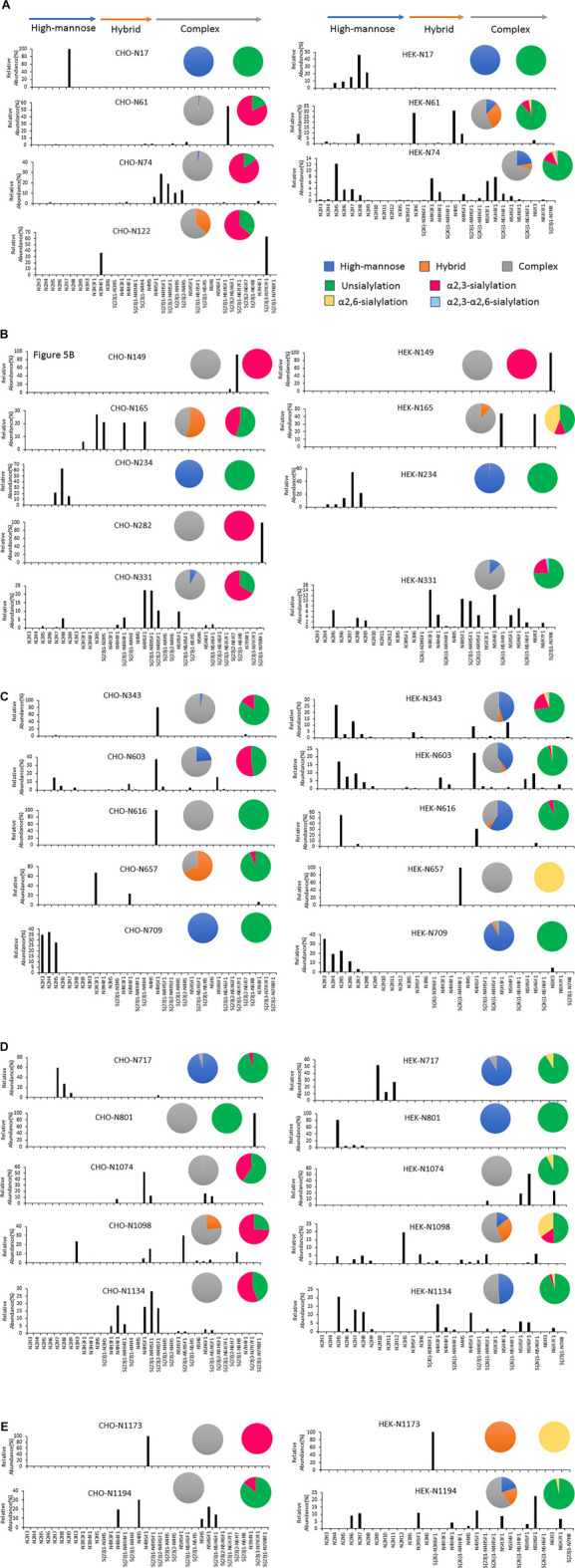
The site-specific major N-glycan distribution and comparison of SARS-COV-2 spike from CHO and HEK cells, respectively. The pie charts summarized the N-glycan type distribution and the sialylation linkage information identified at each site. The key is shown in **(A)**. Glycosite comparisons are shown as follows: **(A)** Glycosites N17, N61, N74 and N122, **(B)** N149, N165, N234, N282 and N331, **(C)** N343, N603, N616, N657 and N709, **(D)** N717, N801, N1074, N1098 and N1134, and **(E)** N1173 and N1194. The complete lists of N-glycans at each site were presented in Supplementary Table S5.

In addition, the distribution of site-specific N-glycan on each identified N-glycosite from CHO and HEK cells is displayed in Figure 5 and compared with the site-specific N-glycan compositions and percentages listed in Supplementary Table S5. Consistent with the global N-glycan analysis, we observed more N-glycan types and more high-mannose content on the S glycopeptides from HEK cells, especially in N17, N234, N343, N616, N709, N801, and N1134 sites. The higher abundance of high mannose glycans we observed at the listed sites may be due to differences in the inherent glycosylation propensities of the 2 cell substrates. It should be noted that the absence of complex N-glycans has been reported to reduce the entry of viruses into HEK-ACE2 expressing cells by more than 95% (Yang et al., 2020). Complex N-glycans mainly exist at the N74, N331, N1074, N1098 and N1194 sites on HEK-Spike. Most spike glycopeptides from HEK cells tend to be unsialylated, with a small amount of α2,3- and α2,6- or both α2,3 and α2,6-sialylation linkage types at the N17, N64 and N343 sites. The analysis of the glycans identified on the native-like SARS-CoV-2 virus revealed a high abundance of high-mannose type and complex N-glycans with low sialylation content (Casalino et al., 2020; Grant et al., 2020; Watanabe et al., 2021). Alternatively, CHO-Spike mainly presents complex N-glycans as the principal glycan form on these and most other sites. Indeed, CHO-Spike is highly sialylated at N61, N74, N343, N1098 and N1134 sites, exclusively containing α2,3-sialylation. Additionally, the level of Neu5Gc detected at the N331 site (0.11% at this site) of spike from CHO cells was negligible. Furthermore, as a heavily glycosylated site on CHO-Spike and HEK-Spike, N74 presents highly α2,3-sialylated complex glycans on CHO-Spike and displays mainly unsialylated complex glycans with some high-mannose and hybrid N-glycans on HEK-Spike. Consequently, our results indicate that the N-glycosylation in full-length spike protein generated by HEK cells, is significantly different from other constructs, such as S1 and S2 independently expressed constructs as well as spike generated from CHO cells. However, separate serum ELISA assay using COVID antigens from either transient HEK and CHO lines revealed that there is no significant difference in the antibody binding response as a function of dilution level, so both antigens may be useful reagents for immunoassay detection (data not shown).

Finally, the S proteins analyzed here were engineered to contain a C-terminal trimerization domain as the transmembrane domain was removed. The trimerization domain was put in place to mimic trimerization in the engineered soluble form that would otherwise occur in the native transmembrane spike. Due to these changes glycosylation patterns may differ from those that would be observed in the TM containing native spike expressed in HEK and CHO cells.

## Conclusion

In this work, recombinant full-length SARS-CoV-2 spike was produced transiently in suspension CHO and HEK hosts. We then provided global and site-specific analysis of N-linked glycosylation on the soluble full-size S glycoprotein. To evaluate the sialic acid linkages present on the spike glycans, a two-step derivatization by dimethylamine and ammonium hydroxide was performed using a solid support system to differentially modify the sialic acid linkages on the resulting glycans and glycopeptides from the two hosts. We identified the glycan compositions at 21 and 19 out of the 22 predicted N-glycosylation sites of the SARS-CoV-2 S proteins in CHO and HEK, respectively. The N-glycan site at 1,158 position (N1158) was found unoccupied on spike from CHO and the N-glycan sites at 122, 282 and 1,158 positions (N122, N282 and N1158) were found unoccupied on spike secreted from HEK cells. Structural mapping of glycans of recombinant full-length human S proteins revealed that CHO-Spike presented more complex and higher sialylation (α2,3-linked only; ∼40% of total) content while HEK-Spike had marginally more high-mannose glycan, almost exclusively as Man_5_GlcNAc_2_, and minor amounts of α2,3- and α2,6 linked sialylation (less than 15% total). The N74 site represents the most heavily and compositionally diverse N-glycosylated site on both spike proteins. The abundant high-mannose sites (N17, N234, N343, N616, N709, N717, N801 and N1134) on HEK-Spike may serve as glycan shields and offer viral evasion properties and/or provide targets for mannose specific immune system lectins (Hsieh et al., 2018; Rahimi, 2020). Alternatively, the complex type N-glycans presenting at N74, N331, N1074, N1098 and N1194 sites on HEK-Spike may facilitate viral entry into ACE2 expressing cells (Yang et al., 2020) as well as serve as glycan shield. Collectively, these data underscore that certain N-glycan sites offer distinctive glycosylation patterns regardless of the host, while other sites exhibit site-specific differences in glycosylation in different mammalian hosts. These distinct site-specific N-glycan profiles may impact viral behavior *in vivo* and perhaps impact vaccine development, and these differences may have no problem or impact on specific diagnostic applications.

## Data Availability

The datasets presented in this study can be found in online repositories. The names of the repository/repositories and accession number(s) can be found below: ProteomeXchange Consortium *via* the PRIDE partner repository with the dataset identifier PXD027536.
