# Supplementary figures and images for: A Linkage-specific Sialic Acid Labeling Strategy Reveals Different Site-specific Glycosylation Patterns in SARS-CoV-2 Spike Protein Produced in CHO and HEK Cell Substrates
Source: Front Chem. 2021 Sep 24;9:735558. doi: 10.3389/fchem.2021.735558 (PMC8497748; doi:10.3389/fchem.2021.735558)

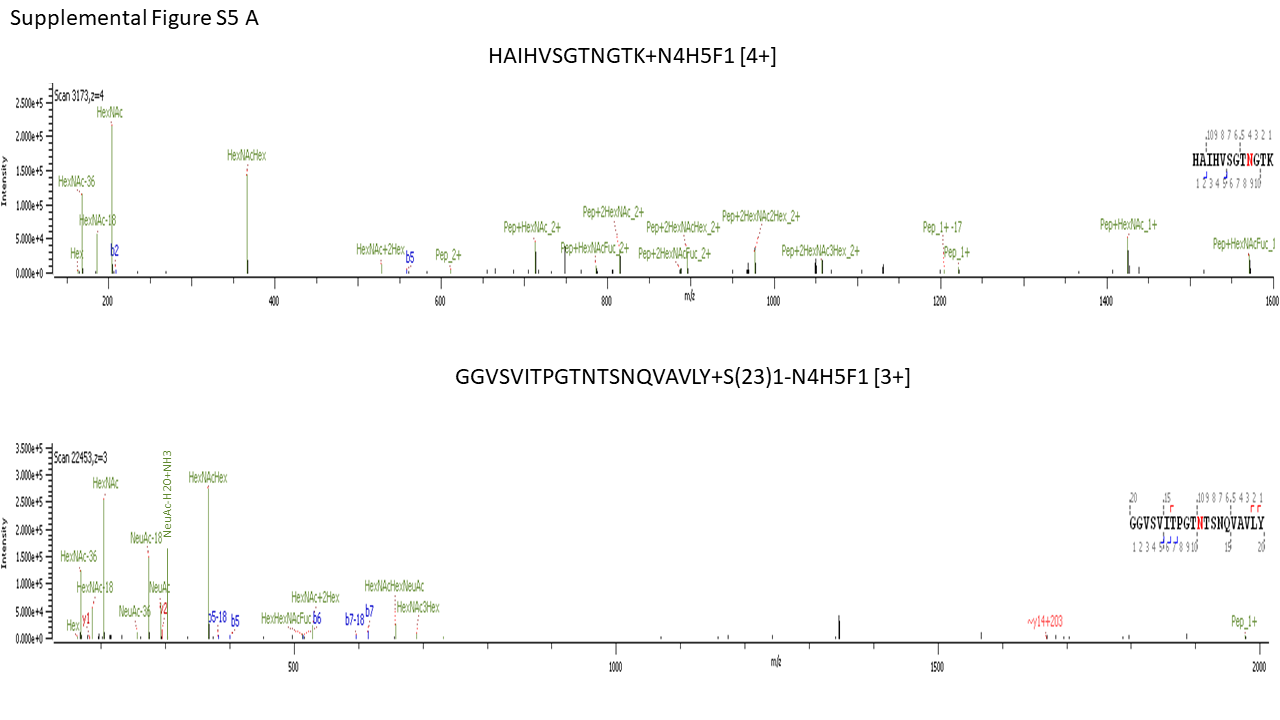

Supplement: Supplementary file 2 [file Image6.TIF]

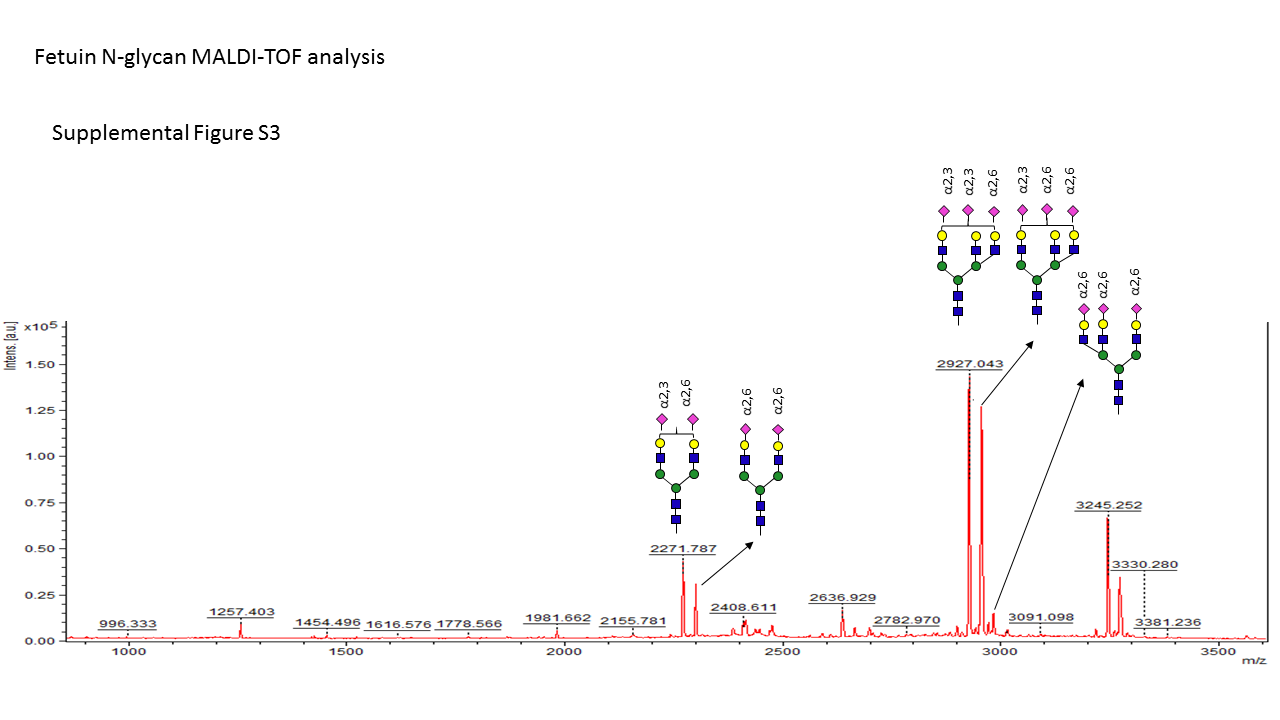

Supplement: Supplementary file 3 [file Image3.TIF]

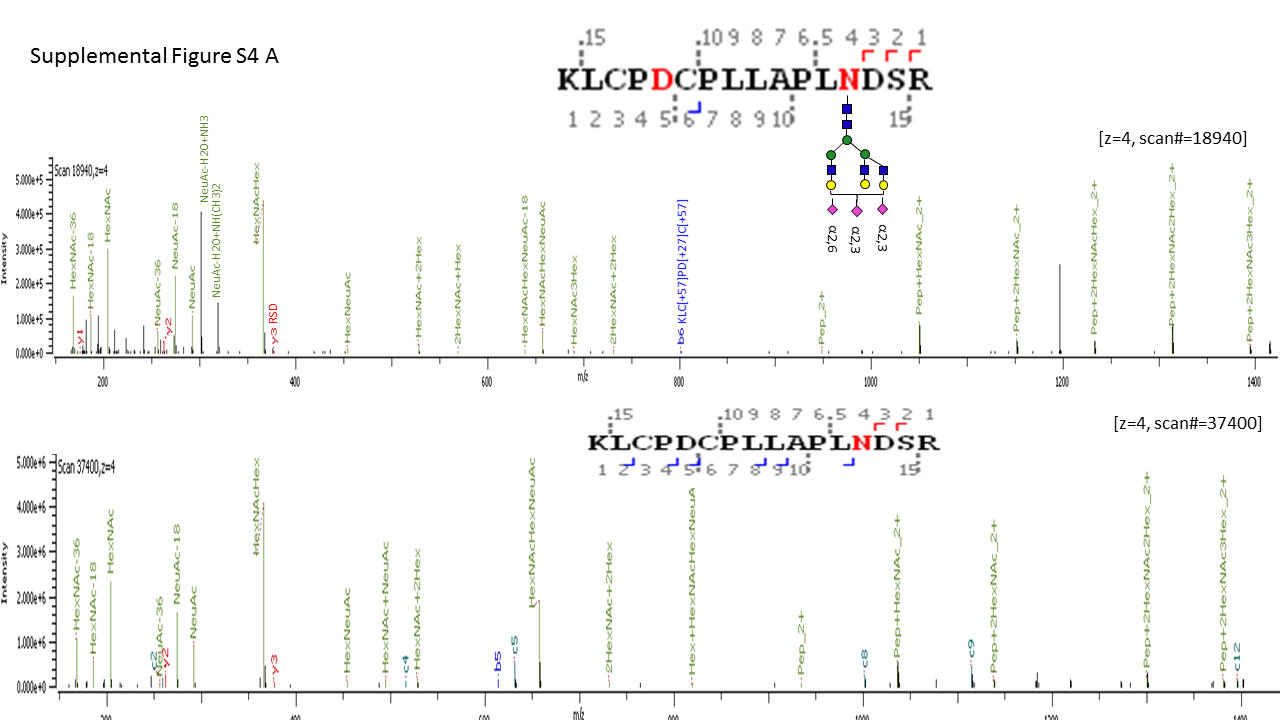

Supplement: Supplementary file 4 [file Image4.TIF]

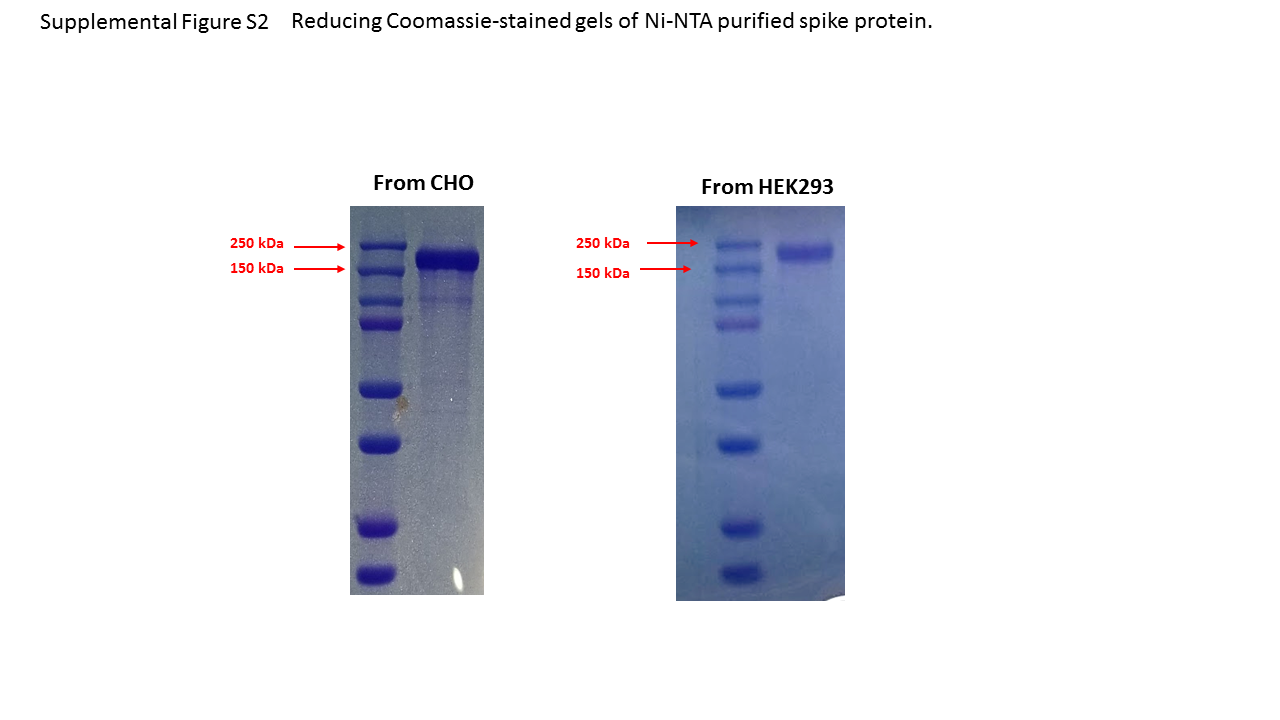

Supplement: Supplementary file 5 [file Image2.TIF]

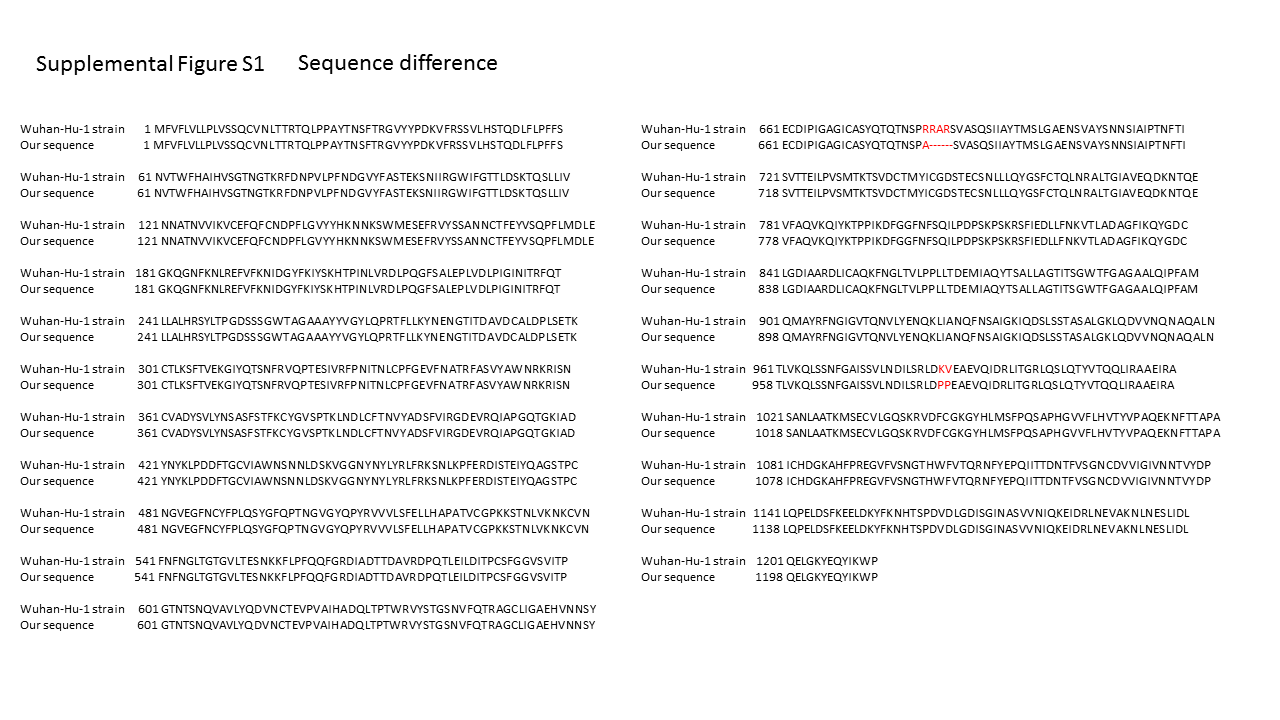

Supplement: Supplementary file 6 [file Image1.TIF]

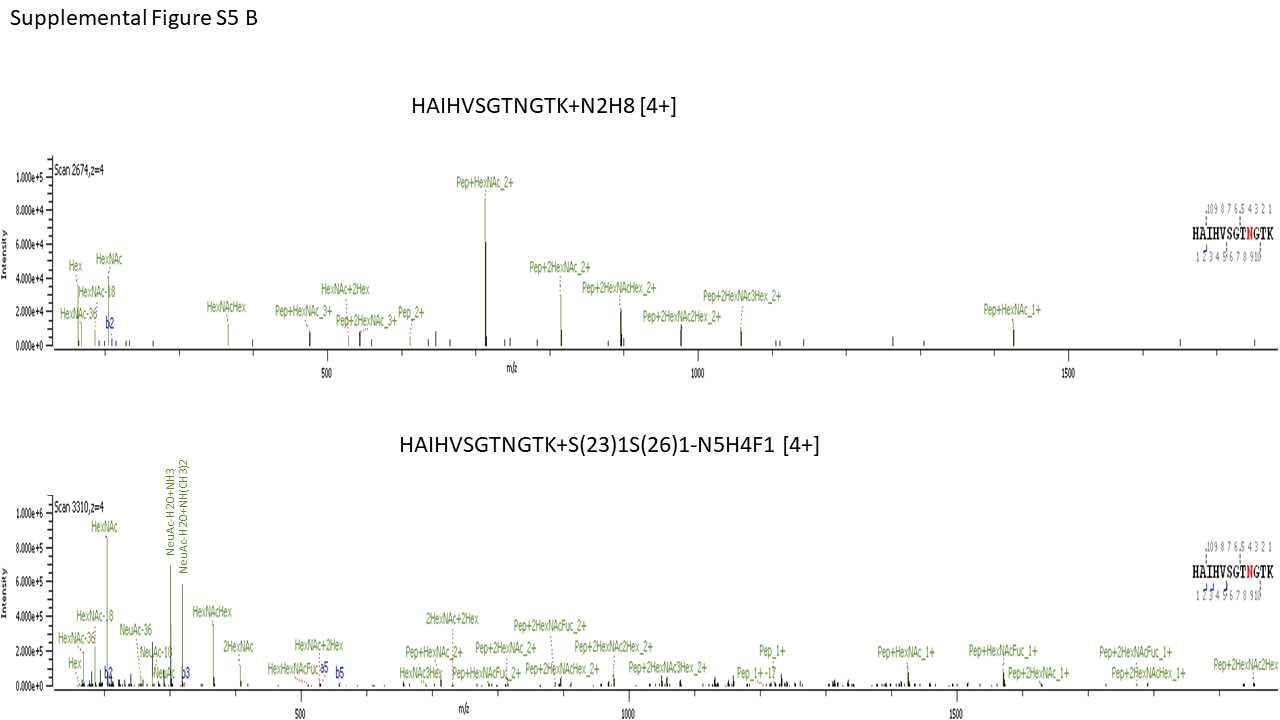

Supplement: Supplementary file 7 [file Image7.TIF]

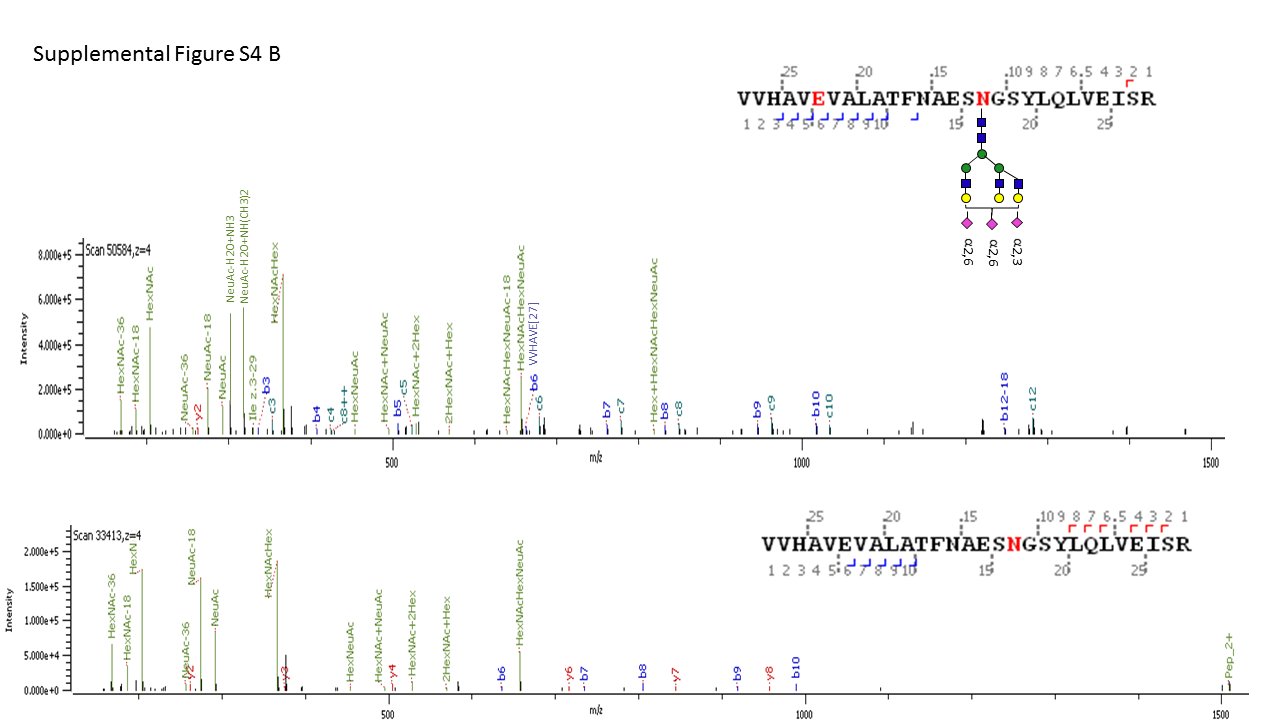

Supplement: Supplementary file 10 [file Image5.TIF]
