# Supplementary material for: A Linkage-specific Sialic Acid Labeling Strategy Reveals Different Site-specific Glycosylation Patterns in SARS-CoV-2 Spike Protein Produced in CHO and HEK Cell Substrates
Source: Front Chem. 2021 Sep 24;9:735558. doi: 10.3389/fchem.2021.735558 (PMC8497748; doi:10.3389/fchem.2021.735558)
Supplement: Supplementary file 8 [file Table3.DOCX]

Supplemental figure and table legends

Supplemental Figure S1. Comparison of the sequence in this work with that of severe acute respiratory syndrome coronavirus 2 isolate Wuhan-Hu-1 (GenBank: MN908947.3).

Supplemental Figure S2. SDS-PAGE Coomassie blue staining of the purified spike proteins from CHO and HEK cells used in this study.

Supplemental Figure S3. MALDI-TOF analysis of bovine fetuin released N-glycan with sialic acids modified by sequential amidation with dimethylamide (Sialylα2,6 label) and ammonium hydroxide (Sialylα2,3 label).

Supplemental Figure S4. Representative MS/MS spectra of intact fetuin glycopeptide with (upper spectra) and without (lower spectra) sialic acid derivation. Upper spectra: Intact glycopeptides containing N156 (S4A) and N176 (S4B) sites of fetuin, modifications of carboxylic acid on D, E amino acids as well as on the sialic acids of N-glycans, lower spectra: unmodified intact glycopeptide with same amino acid sequence.

Supplemental Figure S5. Representative MS/MS spectra of Sialic acid differential labeling intact glycopeptide of CHO-Spike (S5A) and HEK-Spike (S5B).

Supplemental Table 1. Byonic searching parameters.

Supplemental Table 2. The full list of identified N-glycans from fetuin in this work.

Supplemental Table 3. Relative Abundance of modified D and E amino acids by dimethylamidation and amidation, respectively.

Supplemental Table 4. The full list of unmodified and amidated identified bovine fetuin N-glycan compositions and their apparent site-specific percentages of total ion abundances.

Supplemental Table 5. The full list of identified N-glycan compositions with their percentages of total ion abundance at each identified glycosite from CHO-Spike and HEK-Spike in this work.
