# Supplementary material for: A Linkage-specific Sialic Acid Labeling Strategy Reveals Different Site-specific Glycosylation Patterns in SARS-CoV-2 Spike Protein Produced in CHO and HEK Cell Substrates
Source: Front Chem. 2021 Sep 24;9:735558. doi: 10.3389/fchem.2021.735558 (PMC8497748; doi:10.3389/fchem.2021.735558)
Supplement: Supplementary file 11 [file DataSheet1.docx]

Supplemental Table 1.

| Byonic search parameters | N-glycopeptide |
| --- | --- |
| Cleavage site | KR for Fetuin/RKFYW for spike protein |
| Cleavage side | C-terminal |
| Missed cleavage | 2 |
| Precursor mass tolerance | 10 ppm |
| Frgament mass tolerance | 15 ppm for HCD and 15 ppm for EThcD |
| Modifications | Oxidation / +15.994915 @ M \| common2 |
|  | Carbamidomethyl / +57.021464 @ C \| fixed |
|  | DTT / +151.996571 @ C \| common1 |
|  | Dimethalamidation / +27.047 @ Cterm, D, E \| common3 for fetuin, common2 for Spike |
|  | HecNAc/+203.079373 @Nglycan \| common1 |
| Glycan database | mammalian N-glycans with (NH(CH3)2) and NH₄OH labeled on sialic acids@ Nglycans\| common1 |
| Mannual Score cutoff | 50 |
| Protein FDR | 1% |
| Maximum precursor mass | 20000 |
| Precursor and charge assignment | compute from MS1 |
| Maximum # of precurosrs per MS2 | 1 |
| Smoothing width (m/z) | 0.01 |

Supplemental Table S2. Fetuin N-glycans

| Experimental Mass [M+Na]+ | Theoretical Mass [M+Na]+ | Delta Mass | Composition | Relative percentage |
| --- | --- | --- | --- | --- |
| 1257.403 | 1257.428 | -0.025 | N2H5 | 2.08% |
| 1419.455 | 1419.481 | -0.026 | N2H6 | 0.55% |
| 1581.468 | 1581.533 | -0.065 | N2H7 | 0.48% |
| 1622.555 | 1622.56 | -0.005 | N3H6 | 0.49% |
| 1809.552 | 1809.644 | -0.092 | N4H5F1 | 0.52% |
| 1953.613 | 1953.698 | -0.085 | S(23)1-N4H5 | 0.54% |
| 1981.662 | 1981.729 | -0.067 | S(26)1-N4H5 | 1.35% |
| 2243.723 | 2243.809 | -0.086 | S(23)2-N4H5 | 0.84% |
| 2271.787 | 2271.84 | -0.053 | S(23)1S(26)1-N4H5 | 9.63% |
| 2299.816 | 2299.871 | -0.055 | S(26)2-N4H5 | 6.79% |
| 2445.903 | 2445.929 | -0.026 | S(26)2-N4H5F1 | 0.88% |
| 2608.880 | 2608.942 | -0.062 | S(23)2-N5H6 | 0.82% |
| 2636.929 | 2636.973 | -0.044 | S(26)1S(23)1-N5H6 | 2.99% |
| 2664.954 | 2665.004 | -0.049 | S(26)2-N5H6 | 1.39% |
| 2782.970 | 2783.03 | -0.061 | S(26)1S(23)1-N5H6F1 | 0.82% |
| 2810.945 | 2811.061 | -0.117 | S(26)2-N5H6F1 | 0.76% |
| 2898.987 | 2899.053 | -0.066 | S(23)3-N5H6 | 1.50% |
| 2927.043 | 2927.084 | -0.041 | S(26)1S(23)2-N5H6 | 23.67% |
| 2955.127 | 2955.115 | 0.012 | S(26)2S(23)1-N5H6 | 20.09% |
| 2983.162 | 2983.146 | 0.016 | S(26)3-N5H6 | 2.61% |
| 3073.027 | 3073.142 | -0.115 | S(26)1S(23)2-N5H6F1 | 0.70% |
| 3101.137 | 3101.173 | -0.036 | S(26)2S(23)1-N5H6F1 | 0.69% |
| 3217.204 | 3217.22 | -0.017 | S(26)2-N7H6F1 | 1.07% |
| 3245.252 | 3245.226 | -0.026 | S(26)2-N5H6S(23)2 | 9.32% |
| 3270.119 | 3270.173 | -0.054 | N8H9F1 | 0.90% |
| 3273.281 | 3273.257 | -0.024 | S(23)1S(26)3-N5H6 | 5.04% |
| 3301.266 | 3301.288 | 0.022 | S(26)4-N5H6 | 0.66% |
| 3408.222 | 3408.264 | -0.042 | S(26)1S(23)1-N8H7 | 0.44% |
| 3563.399 | 3563.369 | -0.042 | S(23)2S(26)3-N5H6 | 0.50% |
| 3582.302 | 3582.328 | -0.026 | S(26)1S(23)3-N6H7 | 0.34% |

Supplemental Table 3. Relative Abundance of modified D and E amino acids by dimethylamidation and amidation respectively

| Glycopeptides of fetuin | Relative Abundance | | Total Abundance in identified glycopeptides | Missed cleavage |
| --- | --- | --- | --- | --- |
|  | unmodified | Dimethylamidation |  |  |
| KLCPDCPLLAPLNDSR | 3.05% | 11.80% | 14.85% | 0 |
| LCPDCPLLAPLNDSR | 0.69% | 0.00% | 0.69% | 0 |
| PTGEVYDIEIDTLETTCHVLDPTPLANCSVR | 0.30% | 0.38% | 0.68% | 0 |
| RPTGEVYDIEIDTLETTCHVLDPTPLANCSVR | 0.00% | 22.91% | 22.91% | 1 |
| VVHAVEVALATFNAESNGSYLQLVEISR | 0.00% | 60.64% | 60.64% | 0 |
| CDSSPDSAEDVRKLCPDCPLLAPLNDSR | 0.00% | 0.23% | 0.23% | 1 |
